# Applying the Social Cognitive Theory to Design a Health Education Program on Weight‐Loss Behaviors Among Police Officers: A Randomized Controlled Trial

**DOI:** 10.1002/osp4.70044

**Published:** 2025-01-08

**Authors:** Mohsen Saffari, Alireza Esmaeili, Hormoz Sanaeinasab, Hojat Rashidi‐jahan, Fatemeh Rahmati, Chung‐Ying Lin, Mark D. Griffiths

**Affiliations:** ^1^ Health Research Center Life Style Institute Baqiyatallah University of Medical Sciences Tehran Tehran Iran; ^2^ Health Education Department Faculty of Health Baqiyatallah University of Medical Sciences Tehran Iran; ^3^ Institute of Allied Health Sciences College of Medicine National Cheng Kung University Tainan Taiwan; ^4^ Biostatistics Consulting Center National Cheng Kung University Hospital College of Medicine National Cheng Kung University Tainan Taiwan; ^5^ International Gaming Research Unit Psychology Department Nottingham Trent University Nottingham UK

**Keywords:** body mass index, health promotion, police force, social cognitive theory, triglyceride, weight reduction

## Abstract

**Background:**

Being overweight/having obesity is a prevalent condition not only among the general population but also among individuals with special occupations such as police officers, where fitness is often a necessity. The present study's aim was to assess how much a psychoeducational intervention based on social cognitive theory (SCT) would be helpful for encouraging weight loss behaviors among police officers.

**Methods:**

In a randomized control trial, 102 police officers who were overweight or had obesity voluntarily registered for a weight loss program and were assigned to either an intervention or control group. Participants in the intervention group took part in a health education program comprising six face‐to‐face 60–80 minute sessions over a 6‐week period that was designed based on SCT principles. The control group participated in routine worksite health promotion programs comprising weekly one‐hour sessions on lifestyle‐related topics and optional individual nutrition consultations. At baseline and 3 months after the intervention, both groups were examined in terms of SCT's measures and weight control strategies as the primary outcomes, as well as para‐clinical variables such as blood lipid profile and body mass index (BMI) as secondary outcomes. Analysis of covariance was used to assess changes from baseline to follow‐up between groups.

**Results:**

All SCT measures indicated significant improvements among those in the intervention group compared with the control group. Outcome expectancy (*F* = 445; *p* < 0.001) and self‐efficacy (*F* = 366; *p* < 0.001) were the two most influential factors. Although both intervention and control groups reported significant changes in weight control behaviors, all behavioral scores in the intervention group were considerably greater than those in the controls. The dietary choice component showed the greatest change from baseline to follow‐up (*F* = 267; *p* < 0.001). All indices of blood lipid profile also indicated significant improvements in the intervention group compared with the control group. Weight decreased significantly more from baseline to follow‐up among those in the intervention group compared with those in the control group (*F* = 69.0, *p* < 0.001). BMI was reduced in the intervention group, while in the control group showed a slight increase (mean difference: −0.4 [CI: −0.4 to −0.3] versus 0.1 [CI: 0.0 to 0.1]).

**Conclusions:**

An education program based on SCT may contribute to weight loss among police officers who are overweight/have obesity. Assessment of the program's effects in other settings and occupational populations is warranted.

## 1 Introduction

According to anthropometric measures, having a body mass index (BMI) equal to 25–30 kg/m^2^ is defined as being overweight, and a BMI greater than or equal to 30 indicates having obesity [1]. Worldwide obesity has tripled over the past 50 years and it is estimated that there are currently more than two billion adults who are overweight [2, 3]. Moreover, two‐thirds of individuals who are overweight or have obesity live in developing countries where there is no adequate access to preventive measures and healthcare facilities [4]. This may increase the mortality rate (and related complications) among affected individuals. For example, a recent systematic review reported that the prevalence of being overweight or having obesity in Iran (where the present study was carried out) was 20% and 13.4% respectively [5].

Studies have shown that police officers who are overweight or have obesity may experience an impaired reaction time in their occupational tasks that may considerably diminish their work performance [6]. Moreover, the rate of physical activity among these officers is less than adequate and they usually have a dietary calorie intake greater than recommended levels that may particularly worsen their obesity [7]. Police officers often have long working shifts and experience stressful events that may compromise their sleep quality and mental health [8]. These consequences along with being overweight or having obesity may considerably increase the risk of cardiovascular diseases among this population [9].

There are also several studies in different countries (including Iran) that have reported a high prevalence of obesity among police officers, as well as related comorbidities such as hypertension, hyperlipidemia, and metabolic syndrome [5, 8, 10, 11]. For example, a cross‐sectional study in Saudi Arabia (Iran's neighboring country) reported that more than two‐thirds of police officers were overweight or had obesity [9].

To date, a few prevention programs have been developed that target obesity among police officers. In one study, the effectiveness of an exercise program under supervision was assessed on variables including body composition, body fitness, and cardiovascular conditions in a quasi‐experimental study among a sample of police officers who were overweight [12]. The findings showed at both follow‐ups (6 and 12 months after the commencement of the program) that there were significant improvements in BMI, muscular strength, and heart rate reserve. In another study, the effect of a nutritional intervention on body composition among police officers who were overweight was examined and showed that different nutritional treatment modalities may be helpful to reduce weight after 12 weeks [13].

The main limitation of the previously published studies is that all interventions were usually planned under direct supervision of the researchers, and may only be effective when individuals feel themselves to be under supervision. Moreover, several authors have noted that the maintenance of the improvements over time may not be guaranteed [12, 14]. The best way to overcome such limitations is to integrate behavior changes into individuals' lifestyles so that they may adhere to them even after the termination of the study [15]. In other words, the main goal of health education programs is to establish persistent lifestyle changes that may improve the health status and quality of life, particularly among high‐risk populations [16].

To design and apply such programs, empirically‐supported behavior change theories and models should be utilized. Such programs would be helpful because they may provide a robust and testable framework to explain behavior and to direct the development and implementation of the programs effectively [17]. Such theories and models would also aid researchers in finding the most appropriate targets and tools to strengthen the changes and identify the most influential methods for intervention, data collection, and evaluation [18].

Using theory‐based health education programs to address obesity among different populations has been reported in a few studies. For example, Goger and Cevirme designed an intervention program based on the theory of planned behavior (TPB) for 78 women. Those in the intervention group received a 6‐month training program and counseling service based on the TPB. They found that the program was more effective in decreasing anthropometric values and inactivity rate among the intervention group compared with controls [19]. In a systematic review, Hackman and Knowlden reported 10 experimental studies that had used TPB or the related theory of reasoned action to perform dietary interventions for adolescents and young adults, which reported promising results [20]. In another study, Suire et al. used motivational interviewing based on self‐determination theory to address weight management among college students (*N* = 40). They compared two groups, one that received motivational interviewing and the other that received only six monthly online educational sessions. They found that the intervention group showed better body composition maintenance compared with the control group [21].

Social cognitive theory (SCT) is one of the popular behavior change theories that describes the role of individual properties, social influences, and environmental factors in health‐related behaviors [22]. The SCT was initially developed by Albert Bandura in the 1960s as “social learning theory” and includes the reciprocal interaction of individual, environment, and behavior. The theory can help explain the pathways by which individuals acquire and continue their behaviors in the social context [23]. The advantage of SCT compared to other behavior change theories is its focus on the behavior maintenance through control and reinforcement rather than just initiation of the behavior [18]. Consequently, this may be more helpful when addressing a behavior like weight control that should be maintained over time.

Some previous studies have applied SCT to their interventions. Psota et al. used SCT and a problem‐based learning approach to prepare a nutrition education program for women. During a 4‐month period, participants (*N* = 101) were trained to reduce calorie intake to achieve an overall weight loss of 10%, and were then followed up a year later. Despite a significant calorie deficit and diet quality improvement among the intervention group compared with the control group, there were no significant differences between the two groups in either weight loss or fat intake [24]. In another study, SCT was used in a 7‐month school‐based nutrition education program for adolescent girls who were overweight/had obesity (*n* = 172). The results showed a reduction in BMI, waist circumstance, and dietary intake among the intervention group compared with controls. Also, the SCT construct indicated noticeable improvements [25].

Knowlden et al. conducted a study with mothers (*N* = 57) to prevent pediatric obesity among their children using both web‐based education and the reciprocal determinism concept of SCT. They found that both methods were effective in improving physical activity, reducing the consumption of sweet beverages, and decreasing screen time. Moreover, the SCT‐based intervention was also more effective in increasing fruit and vegetable consumption [26]. In addition, a systematic review examining SCT‐based obesity intervention programs for adolescents reported four intervention studies (two randomized control trials [RCTs] and two quasi‐experimental studies) that all demonstrated significant reduction in BMI among the intervention groups compared to controls. However, their impact on the physical activity and diet were mostly non‐significant [27].

Among the few studies that have used SCT as a framework to design an education program in a work setting, Abdi et al. applied SCT alongside new communication technology for weight control among governmental employees in Hamadan (a city in Iran). They used telephone/web‐assisted interventions to educate employees who were overweight or had obesity about healthy lifestyles over a 6‐month period. They reported that telephone‐assisted intervention may be more effective than web‐assisted intervention for weight loss and improvement in the constructs such as self‐efficacy, environment, and outcome expectations/expectancies [28]. Although studies have used SCT as a framework in interventions for reducing weight and obesity, most have focused on the secondary preventive measures. However, it appears that this theory may also be used in studies with emphasis on primary prevention where there is a gap in the literature.

These aforementioned intervention studies (SCT or non‐SCT‐based) indicate that there are no consistent findings regarding their efficacy. Therefore, further investigation is needed to better understand how different psychosocial theories and models may be beneficial in designing intervention programs. Moreover, the target groups in the aforementioned studies were more likely to involve children, adolescents, and young people. Consequently, there is limited evidence on how these interventions may work among adult populations. Moreover, the period of intervention for most of the studies was more than 1 month. Some authors suggest that well‐designed short‐term interventions may also result in effective and relatively long‐lasting effects on weight loss behaviors [29, 30]. However, no previous studies have investigated a psychoeducational program comprising all the main constructs of SCT, and there is also no evidence on the actual effect of such programs in work settings, particularly among workers that require a high demand for fitness such as active military forces and police officers. Another gap regarding the efficacy measurement in prior studies is their focus on the self‐report scales or just including weight and BMI as the primary outcomes for assessment. Therefore, including some para‐clinical measures related to weight loss (e.g., blood lipid profile) may provide a more comprehensive approach for outcome measurements instead of just subjective or easy‐to‐assess measures.

Because SCT may provide appropriate directions on how to plan an intervention program to address police officers who are overweight or have obesity, the present study assessed the efficacy of a relatively brief but well‐designed health education program for weight loss behaviors based on SCT among this population. Because the effectiveness of such interventions has been implicitly demonstrated in the previous studies [20, 31, 32], it was hypothesized that (i) the intervention program would significantly improve behaviors and clinical variables related to weight loss in the intervention group (H_1_), and (ii) the weight loss changes between the intervention and control groups would be significantly different from baseline to follow‐up (H_2_).

## 2 Methods

### 2.1 Participants and Procedure

A randomized controlled trial was conducted at police force headquarters located in Khoy city, one of West Azerbaijan province's cities in the north‐west of Iran. The study was conducted between July and November 2021. Sample size was calculated using the formula suggested by Hulley et al. with the following parameters: *α* (two‐tailed) = 0.05, *β* (power of test) = 0.2, effect size (mean difference) = 0.6, standard deviation = 1, and same number of participants in each group [33]. This meant that the minimum sample size was 51 participants in each group assuming a 10% attrition rate.

Using convenience sampling, all volunteers who were overweight or had obesity were invited to participate through announcements on notice boards located at the worksites of potential participants. The inclusion criteria were: (i) having a BMI greater than 25 kg/m^2^ (according to field measurement), (ii) being an official police officer, and (iii) having police work experience for over 1 year. The exclusion criteria were individuals who (i) had any severe health conditions such as severe hypertension and diabetes mellitus, (ii) were receiving medical treatment for being overweight or related disorders, (iii) took part in any weight‐loss intervention program during the past 6 months, and (iv) had any physical limitations that would inhibit their participation in a lifestyle modification program.

**FIGURE 1:**
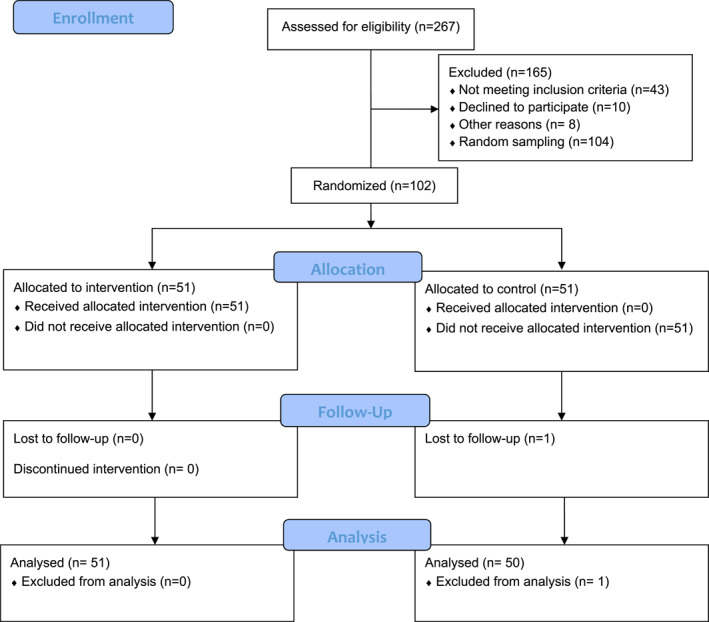
CONSORT flow diagram.

A total of 102 participants were randomly selected from 206 individuals who met the inclusion criteria. For random sampling, the participants were each assigned a number. Then, using the random number function in Microsoft Excel, a subset of individuals was selected to participate (*n* = 102). A simple randomization method (shuffled deck of cards) was used to assign selected participants into intervention or control groups (see CONSORT flow diagram shown in Figure 1). The intervention group received a health education program based on SCT for 6 weeks, while the control group did not receive any intervention designed by the research team. However, they could participate in any available health promotion programs delivered by the worksite, such as weekly lectures provided by healthcare professionals on healthy lifestyle or nutritional consultations provided by a professional nutritionist. The lectures lasted for approximately 1 hour on a specific week day during work hours. The lecturers were selected based on the specialty needed to present different content. There was no compulsion to participate in such programs, but participation had positive scores for their annual career promotion.

Three months after the end of the intervention program, both groups were again assessed in terms of variables listed in the “Measures” section, and any changes in these variables from baseline to follow‐up were identified. The primary outcome variables were SCT constructs, and weight control strategies, whereas the blood lipid profile and BMI were assessed as secondary outcomes. All participants were informed about the process of the study and their rights for participation. Participants signed a written consent letter and the ethical committee of Baqiyatallah University of Medical Sciences approved the study's protocol (#IR.BMSU.REC.1400.068). The study protocol was also registered in the Iranian Registry of Clinical Trials (ID: IRCT20210915052485N1).

### 2.2 Educational Intervention

Participants in the intervention group experienced an education program comprising six weekly face‐to‐face sessions based on SCT constructs. Each session for the intervention group lasted 60–80 min and took place at times different from sessions on the routine program. The facilitator of all sessions was a health education specialist and the suggested dates (program timetable) for running the educational sessions were provided by the session facilitator at the first meeting, which was designed for introducing the program before the main sessions commenced. Also, before beginning the sessions, participants were put into small groups with each one comprising 8–10 individuals. In the first session, a short lecture along with a group discussion was held to address situational perceptions. In this session, statistics on being overweight or having obesity among different groups of populations at international and national levels were provided. The participants were then asked about the current situation of being overweight among police officers compared to the general population or other occupational groups and how these conditions may affect their occupational tasks and job performance.

In the second session, the focus was on the outcome expectations. In this session, the likely advantages of the having normal weight and adequate fitness (particularly in terms of health, social, and occupational perspectives) were sought through brainstorming. This was so that all participants could propose their viewpoints on the matter. The session coordinator (who was a health education specialist) wrote down all viewpoints on the screen and a final conclusion was made based on the most important points provided collaboratively.

The third session emphasized the outcome expectancies. For this, a group discussion was held on the direct and indirect effects of the outcome expectations (generated in the prior session) on participants' personal life. The participants discussed how the expectations would change their individual lives and how much these changes would be important.

The fourth session was devoted to self‐efficacy. In this session, a short (15‐min) video was played. This featured individuals who had previously suffered from obesity and who had now reached a normal weight. The individuals in the video explained the process and how those watching could overcome the likely obstacles to weight loss. Following this, the health educator described the process of weight loss in smaller stages so that by following practical and easy to perform steps, a person can start and follow weight loss strategies more effectively. At the end of this session, participants could provide any previous individual experiences on weight reduction and the likely reason(s) for failures.

The fifth session was on self‐control (goal setting). In this session, the participants were taught how to set goals for changing behaviors toward weight loss. For this, the goals were divided into proximal and distal goals. This comprised the immediate and easy to perform goals for weight loss (proximal goals) and longer and more general goals that need special planning to achieve (distal goals), along with the ways to monitor goal progress and how to set personal incentives to reinforce goal achievement. Participants were then asked to set their own personal goals and a few examples were assessed and modified for the participants.

In the sixth (and final) session, which considered social support as a supplemental construct of SCT, some senior police commanders (who were from workplace authorities) were invited to attend the session. Participants were then asked to provide their expectations on creating a supportive workplace for weight loss programs. After hearing the participant's viewpoints, the commanders explained the likely strategies to facilitate such programs and assigned incentives such as gifts or rewards for those who were able to attain normal weight. A brief description of each session is included in Table 1.

**TABLE 1:** Educational methods used in each session and related SCT constructs.

Session	SCT construct	Educational methods
1	Situational perceptions	A short lecture on obesity prevalence, discussion on the impacts of the conditions on job performance
2	Outcome expectations	Brainstorm on the potential advantages of having normal weight
3	Outcome expectancies	Discussion on the likely impressions of having normal weight in personal life
4	Self‐efficacy	Displaying a short video of role models, describing how to reach weight loss through small steps, providing personal experiences
5	Goal setting (self‐control)	A lecture on how to set proximal and distal goals for weight loss and monitor the progress and reinforcement, practice goal setting
6	Social support	Dialogue between participants and senior commanders to create supportive workplace for weight loss

### 2.3 Measures

#### 2.3.1 Social Cognitive Theory Questionnaire (SCTQ)

The SCTQ was developed by the research team to assess physical activity and dietary aspects of a healthy lifestyle based on selected SCT components. The SCTQ comprises six subscales comprising situational perceptions, outcome expectations, outcome expectancies, self‐efficacy, self‐control (goal setting), and social support. Scale development was conducted in several stages. In the first stage, an item pool was generated based on the prior related scales as well as guidelines provided by Bandura on how to use SCT in health promotion [34], so that all these selected subscales covered the relevant items. Using an expert panel including five health educators, three health psychologists, two nurses, and one epidemiologist, the item pool was investigated. Based on expert's viewpoints, the number of items was reduced from 118 to 87 items.

In the next stage, the content validity index (CVI) and content validity ratio (CVR) of the scale were computed based on formulas proposed by the Lawshe [35]. After removing inappropriate items based on CVI and CVR results, 66 items were retained. For pilot testing of the scale, 10 police officers participated. They were asked to assess the items in terms of understandability and relevancy and provide their suggestions to improve the readability of the scale. Following this, relevant suggestions were addressed and any changes needed were done. The pre‐final version of the scale was then distributed among 30 police officers who were similar to the main sample in terms of inclusion/exclusion criteria but did not participate in the RCT.

To assess internal consistency of the SCTQ, Cronbach's alpha was computed, which ranged from 0.71 to 0.89 for the six subscales. Stability of the measure was also examined through test‐retest correlation coefficient that produced very good reliability over a 2‐week interval (*r* = 0.84). The final version of the SCTQ comprised 64 items: situational perceptions (8 items; e.g., *“Being overweight/obese is a regular condition for all police officers around the world”*); outcome expectations (10 items; e.g., *“Having a regular physical activity program may protect me against being overweight/obese”*); outcome expectancies (10 items; e.g., *“It is important for me to maintain a normal weight through a regular physical activity program”*); self‐efficacy (14 items; e.g., *“I trust to my capabilities to keep a healthy low‐fat diet for a better physical fitness”*); self‐control/goal setting (10 items; e.g., *“I have a plan to do daily physical activities such as walking or aerobics exercises”*); and social support (12 items; e.g., *“There is a healthy food menu in my workplace that helps me to follow a healthy diet”*). All items on all subscales are rated from 1 (*completely disagree*) to 5 (*completely agree*) that generates a total score between 1 and 5 for the whole scale. The score is computed by summing the scores of all items and dividing by 64 (i.e., the total number of items). Individual scores for each subscale are also computed by summing the scores of all items related to that subscale divided by the number of items in the subscale. Higher scores indicate better lifestyle in relation to SCT constructs on each subscale or the whole measure. Although some psychometric properties of the SCTQ were assessed in the present study, further investigation is needed to confirm its validity and reliability through other methods of psychometric assessment using larger samples.

#### 2.3.2 Weight Control Strategies Scale (WCSS)

The WCSS developed by Pinto et al. (28) was used to assess special behaviors that may facilitate weight loss. The scale consists of 30 items comprising four subscales: (i) dietary choices (10 items; e.g., *“I had several servings of fruits and/or vegetables each day”*), (ii) self‐monitoring (7 items; e.g., *“I set a daily calorie goal for myself”*), (iii) physical activity (6 items; e.g., *“I schedule exercise into my day”*), and (iv) psychological coping (7 items; e.g., *“If I overate, I thought about what led to my overeating”*). Items are rated from 0 (*never*) to 4 (*always*). The total score on the WCSS is calculated by adding the score obtained from all items and dividing by 30, resulting in a score from 0 to 4. Every individual subscale also produces a specific score indicating the frequency of those particular behaviors. This score also ranges from 0 to 4 and is calculated in the same way described for the total score (except dividing by the number of items in the subscale) [36]. Following a forward‐backward translation as suggested by Beaton et al. [37], the Cronbach's alpha was 0.86 for the whole scale and alphas ranging between 0.76 and 0.89 for its subscales.

#### 2.3.3 Demographic and Clinical Measures

Demographic information was collected from all participants, including age, marital status, number of children, education level, work experience, economic status, rank, and daily time devoted to moderate to vigorous physical activity. Participants' weight and height were measured using a digital body weight scale (Beurer, BF‐950‐Germany) and wall mounted height gauge (Seca, 206‐Germany). For this, all height and height measurements were performed in the morning by a trained health education specialist and all participants were asked to use the toilet before weight measurement. Participants were also asked to remove clothing such as their coat and shoes to increase accuracy of the measurements. Laboratory tests were used to measure high‐density lipoprotein (HDL‐ mg/dL), low‐density lipoprotein (LDL‐ mg/dL), triglyceride (mg/dL), and cholesterol (mg/dL). These were measured according to guidelines provided for laboratory diagnostics and quality of blood collection [38].

### 2.4 Data Analysis

All data were entered into SPSS for Windows version 26 (IBM Corp. Armonk, NY) for analysis. Statistics such as means and standard deviations (SDs) were used to describe quantitative data, whereas frequencies and percentages were used to describe categorical data. To compare differences between the intervention and control groups in terms of categorical demographic variables and BMI at baseline, chi‐squared tests were used. Changes in SCT constructs as well as weight loss specific behaviors (WCSS) were tested using analysis of covariance (ANCOVA) tests. For this, the requirements of using ANCOVA including homoscedasticity and normal distribution were assessed using Levene and Shapiro‐Wilk tests, respectively. Using a general linear model, the baseline values were considered as covariates, and the follow‐up values and group variables were included as dependent and fixed factors, respectively. Paired sample *t*‐tests were used to assess within‐group differences, and independent sample *t‐*tests were used to assess between‐group differences. A *p‐*value less than 0.05 was used to indicate significant results.

## 3 Results

The mean age of the participants was 29.6 years (SD = 4.4) and all participants were male. More than two‐thirds of both groups (intervention and control) were married and more than half of them had fewer than two children. Less than one‐quarter of the participants had an education level higher than a bachelor's degree, and on average, their work experience was 8.4 years (SD = 3.5). More than three‐quarters of the participants in both groups reported that they did not engage in moderate to vigorous physical activity daily for at least half an hour. Most reported their economic status as less than good (> 80%). The majority of the participants (nearly 80%) also had a rank as a junior officer. Details on the demographics as well as BMI levels are shown in Table 2.

**TABLE 2:** Characteristics of study groups.

Variables	Intervention group (*n* = 51)	Control group (*n* = 51)	Comparison *χ* ^2^ (*p*‐value)
Age			
< 30	35 (68.6)	29 (56.9)	1.51 (0.306)
≥ 30	16 (31.4)	22 (43.1)	
Marital status			
Single	14 (27.5)	12 (23.5)	0.21 (0.650)
Married	37 (72.5)	39 (76.5)	
Number of children			
< 2	27 (52.9)	35 (68.6)	2.63 (0.105)
≥ 2	24 (47.1)	16 (31.4)	
Education			
Undergraduate	44 (86.3)	40 (78.4)	1.07 (0.299)
Graduate	7 (13.7)	11 (21.6)	
Length of service			
< 10	34 (66.7)	32 (62.7)	0.17 (0.679)
≥ 10	17 (33.3)	19 (37.3)	
MVPA (30 min/day)			
Yes	12 (23.5)	9 (17.6)	0.54 (0.463)
No	39 (76.5)	42 (82.4)	
Economic status			
Poor	27 (52.9)	18 (35.3)	4.08 (0.130)
Fair	20 (39.2)	24 (47.1)	
Good	4 (7.8)	9 (17.6)	
Rank			
Junior officer	43 (84.3)	38 (74.5)	1.49 (0.220)
Senior officer	8 (15.7)	13 (25.5)	
BMI (kg/m^2^)			
25–30	38 (74.5)	44 (86.3)	2.24 (0.135)
≥ 30	13 (25.5)	7 (13.7)	

Abbreviations: BMI, body mass index; MVPA, moderate to vigorous physical activity.

Table 3 describes the changes in SCT constructs from baseline to follow‐up in both groups. Of the six constructs assessed, goal‐setting (self‐control) had the highest scores in both groups, and situational perceptions had the lowest score in both groups. All six constructs improved significantly in the intervention group from baseline to follow‐up (*p* < 0.001). While some constructs such as outcome expectations, social support, and situational perceptions improved in the control group, others like self‐efficacy and outcome expectancies showed significant deterioration (*p* < 0.5). The change in the self‐control construct was not significant among controls. Between‐group comparisons also indicated significant improvement in all six constructs among the intervention group compared with the control group. After adjustment of baseline differences using ANCOVA, constructs including outcome expectancies, self‐efficacy, and situational perceptions showed maximum changes from baseline to follow‐up across the groups respectively, whereas the minimum change was related to goal‐setting/self‐control (445 < *F* < 248 vs. *F* = 57).

**TABLE 3:** Changes in the social cognitive theory constructs from baseline to follow‐up.

Measures	Intervention		Control		Difference (95% CI for difference FL—BL)		Difference (95% CI for difference int.—cont.)		
	BL (*n* = 51)	FU (*n* = 51)	BL (*n* = 51)	FU (*n* = 50)					ANCOVA test
	Mean (SD)	Mean (SD)	Mean (SD)	Mean (SD)	Intervention	Control	Baseline	Follow‐up	F (*p* value)
Self‐efficacy	2.6 (0.7)	3.4 (0.4)	2.3 (0.6)	2.2 (0.5)	0.8 (0.6 to 0.9)**	−0.1 (−0.0 to −0.1)**	0.3 (0.0 to 0.5)*	1.1 (0.9 to 1.1)**	365.9 (< 0.001)
Outcome expectations	2.3 (0.6)	3.3 (0.5)	2.2 (0.8)	2.4 (0.7)	1.0 (0.8 to 1.1)**	0.2 (0.1 to 0.3)**	0.1 (0.4 to −0.1)	0.9 (0.6 to 1.2)**	151.4 (< 0.001)
Outcome expectancies	2.6 (0.6)	3.9 (0.5)	2.4 (0.6)	2.3 (0.5)	1.3 (1.1 to 1.4)**	−0.1 (−0.2 to −0.0)*	0.2 (0.4 to −0.0)	1.6 (1.3 to 1.7)**	444.7 (< 0.001)
Goal setting (self‐control)	3.5 (0.9)	4.1 (0.8)	3.8 (0.8)	3.8 (0.7)	0.6 (0.4 to 0.7)**	−0.0 (−0.1 to 0.0)	−0.3 (0.1 to −0.6)	0.3 (0.2 to 0.6)*	57.3 (< 0.001)
Social support	2.9 (0.6)	3.6 (0.6)	2.7 (0.7)	2.8 (0.6)	0.7 (0.6 to 0.8)**	0.1 (0.0 to 0.1)*	0.2 (−0.1 to 0.4)	0.8 (1.0 to 0.5)**	163.7 (< 0.001)
Situational perceptions	2.1 (0.7)	2.8 (0.6)	2.2 (0.6)	2.3 (0.6)	0.7 (0.6 to 0.8)**	0.1 (0.0 to 0.1)*	−0.1 (−0.4 to 0.1)	0.5 (0.3 to 0.7)**	248.2 (< 0.001)

Abbreviations: ANCOVA, analysis of covariance; BL, baseline; CI, confidence interval; Cont., control group; F, F statistic; FU, follow‐up; Int., intervention group; M, mean; SD, standard deviation.

*p < 0.05; **p < 0.001.

Findings relating to facilitating behaviors of weight loss as well as paraclinical indices are shown in Table 4. Psychological coping obtained the lowest score in both groups, while self‐monitoring in the intervention group and dietary choices in the control group showed the highest score at baseline. However, within‐group comparisons showed significant improvement of all weight loss behaviors in both groups (*p* < 0.001). While, there was no significant difference between groups at baseline except for self‐monitoring and psychological coping (*p* < 0.05), all the behaviors improved significantly in the intervention group compared to controls from baseline to follow‐up (*p* < 0.001). The largest change in weight loss behavior after baseline adjustment was related to dietary choices (*F* = 267, *p* < 0.001) and the least was for self‐monitoring (*F* = 26, *p* < 0.001).

**TABLE 4:** Differences between the groups (intervention vs. control) in weight control strategies scale (WCSS) and clinical characteristics before and after the intervention.

Measures	Intervention		Control		Difference (95% CI for difference FL—BL)		Difference (95% CI for difference Int.—cont.)		
	BL (*n* = 51)	FU (*n* = 51)	BL (*n* = 51)	FU (*n* = 50)					ANCOVA test
	Mean (SD)	Mean (SD)	Mean (SD)	Mean (SD)	Intervention	Control	Baseline	Follow‐up	F (*p* value)
WCSS (Dietary choices)	1.6 (0.6)	3.6 (0.7)	1.7 (0.5)	1.9 (0.4)	2.0 (1.7 to 2.2)**	0.2 (0.1 to 0.2)**	−0.1 (−0.3 to 0.1)	1.7 (1.5 to 1.9)**	266.9 (< 0.001)
WCSS (Self‐monitoring)	1.9 (0.9)	2.4 (0.6)	1.6 (0.6)	1.8 (0.7)	0.5 (0.3 to 0.6)**	0.2 (0.1 to 0.3)**	0.3 (0.0 to 0.6)*	0.6 (0.4 to 0.9)**	25.5 (< 0.001)
WCSS (Physical activity)	1.8 (0.9)	2.9 (0.6)	1.6 (0.7)	1.9 (0.5)	1.1 (0.9 to 1.3)**	0.3 (0.2 to 0.4)**	0.2 (−0.1 to 0.5)	1.0 (0.7 to 1.2)**	111.0 (< 0.001)
WCSS (Psychological coping)	1.3 (0.6)	2.4 (0.6)	1.5 (0.6)	1.9 (0.5)	1.1 (0.9 to 1.2)**	0.4 (0.3 to 0.4)**	−0.2 (−0.5 to −0.0)*	0.5 (0.2 to 0.7)**	84.5 (< 0.001)
WCSS (Total)	1.6 (0.4)	2.8 (0.3)	1.6 (0.4)	1.9 (0.4)	1.2 (1.1 to 1.3)**	0.3 (0.2 to 0.3)**	0.0 (−0.1 to 0.2)	0.9 (0.8 to 1.1)**	600.0 (< 0.001)
HDL (mg/dL)	46.6 (10.6)	49.0 (10.8)	46.9 (12.6)	47.3 (12.2)	2.4 (1.9 to 3.0)**	0.4 (−1.4 to 2.2)	−0.3 (−4.8 to 4.3)	1.7 (−2.8 to 6.3)	4.6 (0.035)
LDL (mg/dL)	124.1 (27.7)	115.6 (24.3)	120.8 (28.7)	122.6 (28.5)	−8.5 (−10.7 to −6.2)**	1.8 (−0.2 to 3.8)	3.3 (−7.8 to 14.4)	−7.0 (−17.4 to 3.4)	49.5 (< 0.001)
Triglyceride (mg/dL)	176.4 (49.3)	167.1 (46.1)	176.8 (44.9)	178.3 (43.3)	−9.3 (−13.7 to −4.9)**	1.5 (−0.3 to 3.3)	−0.4 (−18.9 to 18.2)	−11.2 (−29.0 to 6.3)	23.5 (< 0.001)
Cholesterol (mg/dL)	203.4 (34.5)	191.7 (32.3)	200.3 (38.9)	203.3 (40.5)	−11.7 (−14.1 to −9.4)**	3.0 (1.4 to 4.5)**	3.1 (−11.3 to 17.6)	−11.6 (−26.0 to 2.7)	109.7 (< 0.001)
Weight (kg)	87.5 (9.4)	86.4 (8.9)	86.0 (10.1)	86.3 (10.2)	−1.1 (−1.4 to −0.84)**	0.3 (0.0 to 0.4)*	1.5 (−5.3 to 2.3)	0.1 (−3.9 to 3.6)	69.0 (< 0.001)
BMI (kg/m^2^)	28.5 (2.1)	28.1 (1.9)	28.2 (2.0)	28.3 (2.1)	−0.4 (−0.4 to −0.3)**	0.1 (0.0 to 0.1)*	0.3 (−0.6 to 1.1)	−0.2 (−1.0 to 0.6)	76.4 (< 0.001)

Abbreviations: ANCOVA, analysis of covariance; BL, baseline; BMI, body mass index; CI, confidence interval; Cont., control group; F, F statistic; FU, follow‐up; HDL, high‐density lipoprotein; Int., intervention group; LDL, low‐density lipoprotein; M, mean; SD, standard deviation.

*p < 0.05; **p < 0.001.

Among the paraclinical indices, there was no significant difference between the groups at baseline. However, all these indices improved within the intervention group from baseline to follow‐up (unlike the control group). Although the differences between groups after the intervention did not show any significant change in terms of these indices, adjustment of baseline differences showed that the cholesterol was the most improved variable, and that HDL was the least improved (*F* = 110, *p* < 0.001 vs. *F* = 4.6, *p* < 0.05). Weight decreased significantly more from baseline to follow‐up among those in the intervention group compared with those in the control group when adjustment was conducted for baseline data (*F* = 69.0, *p* < 0.001). The BMI in the intervention group was reduced significantly after the intervention program, whereas BMI showed a significant increase in the control group (*p* < 0.05; mean difference = 0.1, CI: 0.0–0.01).

## 4 Discussion

The present study is the first to examine whether a theory‐based health education program is helpful in promoting weight loss behaviors and weight reduction among a sample of police officers who were overweight or had obesity. It was found that the program was effective both in changing the psychosocial constructs of SCT, as well as the behaviors and objective variables regarding weight loss. In sum, police officers who engaged in the SCT program experienced changes in their psychosocial perspectives on the importance and necessity of weight loss that appear to have helped them toward engaging in healthy behaviors such as having healthy dietary choices, being more physically active, using psychological coping to overcome likely barriers on weight loss, and addressing monitoring behaviors that may contribute to weight loss (confirming both H_1_ and H_2_). All these behaviors may be considered as potential factors that contribute to weight loss and improvements shown in the related laboratory tests.

Several studies using theory‐based interventions for weight loss have been reported, but these have mostly focused on the general population. For example, Gorin et al. [39] in an RCT assessed an intervention program based on self‐determination theory to promote weight loss among 64 couples. They assigned couples into either a standard routine intervention or a particular program to bolster autonomy support as one of the central components of the theory. The program comprised weekly sessions for 6 months and followed the participants for 1 year. Both groups experienced significant weight loss. However, there were no significant differences between the groups. Moreover, those who had lower autonomy support at baseline showed higher weight loss at the final assessment. Therefore, the intervention was effective in improving autonomy support. Since this component was somewhat related to self‐efficacy assessed in the present study, these findings support the idea that if individuals can trust themselves in their abilities to follow weight loss behaviors, and in an environment supportive of such trust, this appears to facilitate the change.

In another study which used a theory‐based intervention, Mirkarimi et al. [40] examined the effect of motivational interviewing individually and combined to an intervention that reinforced intention based on protection motivation theory (PMT). The main premise of this theory is that individuals usually protect themselves using threat and coping appraisals. Therefore, before involving themselves in any preventive behavior, individuals first assess how serious the threat is and how it should be responded. In this theory, self‐efficacy also plays a dominant role and indicates how much an individual may be hopeful in reaching success [41]. Using an RCT design, the authors randomized 150 women who were overweight or had obesity into three groups comprising a standard program, motivational interviewing (MI) program, and MI along with intention intervention program. Four one‐hour sessions were held for those in the standard group on nutritional and psychological aspects of weight loss. Five additional sessions were held for those in the MI group by a psychologist along with a pamphlet prepared based on PMT. Moreover, a detailed timeline on dietary modifications along with prior interventions was considered for the third group (MI plus intention intervention). They reported that compared to the standard group, both intervention groups attained higher scores at a 2‐month follow‐up on PMT constructs, including susceptibility, severity, rewards, self‐efficacy, response efficacy, and costs. However, all groups showed significant but different degrees of weight loss.

Although, Mirkarimi et al.’s study is somewhat relevant to the present study in terms of conducting a theory‐based intervention program and improvement of self‐efficacy, there are some differences that should be addressed. They (i) claimed that they used a theory‐based intervention but only one component of the PMT was (briefly) addressed in their intervention. However, in the present study, an intervention program was developed that included all of the most important components of SCT, and was therefore a more comprehensive approach for a theory‐based intervention; and (ii) had a shorter follow‐up period (2 months compared to 3 months in the present study). The present study also assessed more objective variables including paraclinical tests. Nevertheless, as suggested by other similar theory‐based programs, using such programs is more effective than traditional approaches (such as providing a lecture or pamphlet) on weight loss management [31, 42, 43].

Among the few applications of SCT for weight loss programs, Young et al. [44] applied SCT to assess physical activity (PA) among men who were overweight or had obesity. They aimed to determine which SCT variables may be more helpful in increasing PA. They examined 204 men with an average BMI of 33.1 kg/m^2^ (SD, 3.5) in a longitudinal study. The selected constructs were self‐efficacy, intention, outcome expectations, and social support. They found that self‐efficacy explained a greater part of the variance of PA than other constructs. However, other components were also significant predictors of PA. Therefore, consistent with the present study's findings, they concluded that SCT may be a good theory to predict PA for weight loss among such populations. As in the present study, self‐efficacy was one of the most influential components of SCT contributing to substantial weight loss that could increase PA (assessed in current study using the WCSS).

Another point that merits attention in the present study’s findings is that despite some slight improvements found in the weight loss behaviors among participants in the control group (that may mostly be associated with the changes of their perceptions over time, so that they viewed themselves as more activated on such behaviors at the second assessment), objective measures such as BMI and laboratory tests did not confirm such improvements. This may reflect the fact that many individuals who are overweight or have obesity may actually be interested in weight loss and behavioral modifications toward losing their weights, but their personal viewpoints may deviate across time and may not be confirmed using objective measures. Therefore, using objective measurements (as conducted in the present study) is more helpful in detecting such deviations and provides a more accurate profile of changes experienced through intervention programs.

Although the control group reported an increase in weight loss behaviors, it also had an increased BMI. The difference between an individual's understanding of weight loss behaviors and objective measures supporting these perceptions on weight loss has also been reported in previous studies. For example, Yang et al. examined BMI self‐perception and actual weight management among emerging adults. They found that while nearly 35% of participants were overweight or had obesity, approximately a quarter did not have an accurate perception of their weight, and reported themselves as being of normal weight [45]. In another study, Ishikawa et al. found that both males and females with higher BMI significantly underestimated their body fitness compared with actual anthropometric measures. These findings show that individuals who are overweight or have obesity may have impaired perceptions regarding their weight that may negatively impact the behaviors needed to get fit [46].

Despite the strengths of using an RCT design and conducting a comprehensive intervention program based on SCT's central components, the present study had some limitations that should be noted. First, because the majority of police officers in Iran are males (and although there are no restrictions in being female police officers), there were no females in the sample. Therefore, the findings cannot be generalized to female police officers and further assessment of the effects of such programs in this group is needed. Second, the present study used a convenience sample of officers who were overweight or had obesity, and only individuals who were interested in participating in the present study were enrolled. Therefore, those motivated enough to participate in the study may have different characteristics than those who were not interested in participating. Again, this also reduces the generalizability of findings. Third, the present study only had a 3‐month follow‐up to detect the effects of the program. However, it has been suggested by behavioral scientists that persistence of behavioral changes is more important than initial changes in behavior. However, the sample could not be followed up for longer periods due to limitations in time, resources, and organizational support.

Fourth, long‐term interventions for weight reduction are more likely to be effective than short‐term interventions, as in the present study. However, due to a combination of limited financial resources to run a long‐term intervention as well as the likelihood of the participants not being able to complete the intervention due to job constraints of those working in a military police context, the present study implemented a brief but well‐designed theory‐based intervention to address weight loss behaviors among the target population. However, future studies should consider (where practical) replication over a longer time period. Fifth, no data were collected on the quality or quantity of educational sessions held for the control group or the rate of participation. Therefore, the present study was unable to directly compare the special intervention program with the routine program of the control group.

Finally, the authors did their best to address the most important SCT components as well as the most practical objective measures. However, there are other components that to mitigate the costs, facilitate measurement, and prevent numerous items in the scale, were not included (e.g., knowledge and emotional coping in SCT, and objective variables such as determining the body composition and anthropometric factors).

## 5 Conclusions

The present study demonstrated that using a program based on SCT to educate police officers who were overweight or had obesity appears to be helpful and may motivate this group in relation to weight loss management. Therefore, substituting traditional/standard approaches with theory‐oriented programs may be more effective and provide considerable beneficial changes. Because other occupations who are at risk of sedentary behaviors (e.g., administrative staff, transport drivers) as well as general populations who are also at risk of being overweight or having obesity, performing such interventions in other settings and among individuals with different jobs may provide more evidence on how much such programs may be useful and which psychological variables should further be targeted.

## Author Contributions

M.S. and A.E. both contributed to the conceptualization and preparation of the research proposal as well as data collection and analysis. H.S., H.R.‐J. and F.R. provided suggestions for writing the research proposal and methodology of the study, and also supervised the study. C.‐Y.L. and M.D.G. assessed the manuscript in terms of scientific writing and performed a comprehensive critical review. M.D.G. also helped with language editing of the manuscript and provided constructive comments and revisions to address the reviewer's comments and queries. All authors reviewed the manuscript and cooperated in its writing.

## Conflicts of Interest

The authors declare no conflicts of interest.
